# Paternal Induction of Hybrid Dysgenesis in *Drosophila melanogaster* Is Weakly Correlated with Both *P*-Element and *hobo* Element Dosage

**DOI:** 10.1534/g3.117.040634

**Published:** 2017-03-17

**Authors:** Satyam P. Srivastav, Erin S. Kelleher

**Affiliations:** * Department of Biology and Biochemistry, University of Houston, Texas 77204; † Department of Entomology, Texas A&M University, College Station, Texas 77843

**Keywords:** gonadal atrophy, transposable element, regulation of transposition

## Abstract

Transposable elements (TEs) are virtually ubiquitous components of genomes, yet they often impose significant fitness consequences on their hosts. In addition to producing specific deleterious mutations by insertional inactivation, TEs also impose general fitness costs by inducing DNA damage and participating in ectopic recombination. These latter fitness costs are often assumed to be dosage-dependent, with stronger effects occurring in the presence of higher TE copy numbers. We test this assumption in *Drosophila melanogaster* by considering the relationship between the copy number of two active DNA transposons, *P*-element and *hobo* element, and the incidence of hybrid dysgenesis, a sterility syndrome associated with transposon activity in the germline. By harnessing a subset of the *Drosophila* Genetic Reference Panel (DGRP), a group of fully-sequenced *D. melanogaster* strains, we describe quantitative and structural variation in *P*-elements and *hobo* elements among wild-derived genomes and associate these factors with hybrid dysgenesis. We find that the incidence of hybrid dysgenesis is associated with both *P*-element and *hobo* element copy number in a dosage-dependent manner. However, the relationship is weak for both TEs, suggesting that dosage alone explains only a small part of TE-associated fitness costs.

TEs are ubiquitous genomic parasites that impose a diversity of fitness costs on their hosts. Individual TE insertions are often deleterious because they disrupt functional sequences (Cooley *et al.* 1988; Dupuy *et al.* 2001). However, TEs also contribute to genomic instability by inducing DNA damage during mobilization, and producing structural mutations through ectopic recombination [reviewed in Hedges and Deininger (2007)]. Because the TE-encoded enzymes can catalyze the transposition of any element containing the required *cis*-regulatory sequences, TE-induced DNA damage is expected to be a cumulative effect of all insertions from the same TE family or even multiple related families [reviewed in Hancks and Kazazian (2010) and Fattash *et al.* (2013)]. Similarly, any pair of TE insertions that share sequence homology can potentially undergo ectopic recombination, meaning that the likelihood of ectopic exchange increases with TE copy number (Montgomery *et al.* 1987; Langley *et al.* 1988). Therefore, TEs are assumed to contribute to genome instability in a dosage-dependent manner. However, due to the challenge of quantifying both TE copy number and the associated fitness cost, this assumption is only rarely examined (Bingham *et al.* 1982; Rasmusson *et al.* 1990; Pasyukova *et al.* 2004).

Hybrid dysgenesis syndromes offer a unique opportunity to quantify dosage-dependent fitness effects of TEs. Most extensively studied in *Drosophila*, hybrid dysgenesis is a sterility syndrome caused by unrestricted mobilization of several individual TE families in the germline. Because the germline activities of many TEs are regulated by maternally-deposited Piwi-interacting RNAs (piRNAs), hybrid dysgenesis is observed only among the F1 offspring of certain crosses between males bearing many functional copies of the causative TE and females that do not produce piRNAs to regulate that TE (Brennecke *et al.* 2008). In *Drosophila melanogaster*, the mobilization of three TE families has been associated with hybrid dysgenesis: the DNA transposons *P*-element and *hobo*-element (Bingham *et al.* 1982; Rubin *et al.* 1982; Blackman *et al.* 1987; Yannopoulos *et al.* 1987), and the retrotransposon *I*-element (Bucheton *et al.* 1984).

*P*-element hybrid dysgenesis is a particularly well-studied syndrome with easily quantified fitness effects [reviewed in Kelleher (2016)]. The genomic instability that occurs under high levels of *P*-element activity causes a loss of germline cells, a severe fitness cost that is easily detected and quantified by the atrophied state of the gonads (Schaefer *et al.* 1979; Khurana *et al.* 2011). Among strains harboring *P*-elements, paternal induction of hybrid dysgenesis exhibits continuous variation (Kocur *et al.* 1986; Anxolabéhère *et al.* 1988; Ronsseray *et al.* 1989). However, evidence that this relationship is dosage-dependent on paternal *P*-element copy number is equivocal. While some studies observe modest correlations between the dysgenic phenotype and paternal *P*-element dosage (Bingham *et al.* 1982; Boussy *et al.* 1988), others find that these two variables are uncorrelated (Rasmusson *et al.* 1990; Itoh and Boussy 2002). However, the absence of a strong dosage-dependent relationship may reflect a lack of statistical power, as these analyses relied on Southern blotting to quantify *P*-element copy number among relatively small numbers of strains.

Structural and functional variation among *P*-elements may also confound the relationship between copy number and hybrid dysgenesis. *P*-elements are represented by full-length copies, which encode the transposase enzyme that catalyzes mobilization, and internally-deleted variants that do not encode transposase (O’Hare and Rubin 1983; Sakoyama *et al.* 1985). Although deletion derivatives may be mobilized in the presence of transposase (Karess and Rubin 1984), they also titrate the transposase enzyme that is furnished by full-length elements (Simmons and Bucholz 1985; Rasmusson *et al.* 1990). Additionally, some deletion derivatives, such as the *KP* element, encode truncated transposase proteins that repress *P* activity (Robertson and Engels 1989; Gloor *et al.* 1993; Simmons *et al.* 2002), and may reduce the risk of hybrid dysgenesis (Black *et al.* 1987; Jackson *et al.* 1988).

In this study, we take advantage of the DGRP, a group of fully-sequenced isofemale lines of *D. melanogaster* (Mackay *et al.* 2012), to test the hypothesis that the fitness effects of hybrid dysgenesis are dosage-dependent on *P*-element copy number. We combine novel approaches for detecting and quantifying TE deletion derivatives from deep sequencing data, with previously published TE annotations (Zhuang *et al.* 2014; Rahman *et al.* 2015), to reveal *P*-element copy differences and structural variation among 52 DGRP genomes. For 33 of these strains, we also measure phenotypic variation in paternal induction of hybrid dysgenesis. We demonstrate that although paternal induction of hybrid dysgenesis is highly heritable, it is only weakly associated with paternal *P*-element copy number, even after accounting for potential differences between full-length elements and deletion derivatives. Unexpectedly, we also observe that paternal induction of hybrid dysgenesis is weakly associated with the genomic abundance of *hobo* elements. Indeed, statistical models that consider both *hobo* and *P*-element abundance explain the most variation in induction potential. Collectively, our observations suggest that the fitness costs of hybrid dysgenesis are not strongly dosage-dependent on the causative TE(s), but may be cumulative across multiple recently invaded TE families.

## Materials and Methods

### Estimating P-element and hobo abundance by sequencing coverage

We used previously published paired-end deep sequencing libraries from 52 DGRP strains (Mackay *et al.* 2012) to calculate the relative sequencing coverage of the full-length *P*-element and *hobo* element consensus sequence compared to the *D. melanogaster* reference genome and *hobo* for each sequencing library. These 52 DGRP strains were selected because *P*-elements have previously been annotated in their genomes with the TEMP package (Zhuang *et al.* 2014), allowing us to estimate TE copy numbers using multiple different approaches. Sequences were aligned to release 6 of the *D. melanogaster* reference genome (Hoskins *et al.* 2015) and to the *D. melanogaster P*-element and *hobo* element consensus sequences deposited in FlyBase (www.flybase.org), using HISAT2 with default parameters (Kim *et al.* 2015). For each genome, we estimated the relative abundance of *P*-element and *hobo* element-derived sequences, as compared to single copy sequences, by dividing the average sequencing coverage across the TE by the average sequencing coverage across the reference genome (143.9 Mb, Supplemental Material, Table S1 and Table S2).

### Estimating TE dosage from previous annotations

We also estimated *P*-element and *hobo* element copy number based on previously published TIDAL and TEMP annotations (Zhuang *et al.* 2014; Rahman *et al.* 2015; Table S1 and Table S2). From each annotation set, we calculated two estimators of TE abundance. First, we calculated the total number of annotated *P*-elements or *hobo* elements in each DGRP genome (TIDAL-total and TEMP-total). Second, because TE insertions are often polymorphic even within inbred lines (Zhuang *et al.* 2014; Rahman *et al.* 2015), we estimated the average *P*-element or *hobo* element copy number for an individual from each strain (TIDAL-weighted and TEMP-weighted). To estimate the average copy number, each annotated element was weighted according to its frequency among chromosomes for a given strain, as reported previously by the TEMP and TIDAL packages (Zhuang *et al.* 2014; Rahman *et al.* 2015).

### Estimating the proportion of full-length elements and deletion derivatives

To identify deletion derivatives and estimate their abundance in DGRP genomes, we employed multiple software packages that are designed to assemble transcripts from RNA sequencing data by: (1) detecting novel splice variants, (2) aligning spliced reads, and (3) assembling the alignments. We modified parameters as necessary to eliminate the preference for splice site consensus sequences, thereby allowing the software to detect and assemble deletion derivatives from genome resequencing data. We employed three different combinations of software packages for breakpoint identification and read alignment, and assembled each set of reported alignments using two separate assemblers, producing a total of six assemblies of structural variants for each DGRP genome (Table 1).

**Table 1:** Software packages used to identify and quantify structural variation

Abbreviation	Breakpoint Identification	Alignment	Assembly
gsnap/Cufflinks	gsnap	tophat	Cufflinks
gsnap/StringTie	gsnap	tophat	StringTie
STAR/Cufflinks	STAR	STAR	Cufflinks
STAR/StringTie	STAR	STAR	StringTie
HISAT/Cufflinks	HISAT2	HISAT2	Cufflinks
HISAT/Stringtie	HISAT2	HISAT2	Stringtie

An abbreviation is indicated for each of the six structural variant assemblies generated, as well as the software packages used for each step.

Our three approaches for identification of deletion derivatives and read alignment were as follows. In one approach, which we implemented for *P*-elements only, we aligned reads to the full-length consensus sequence using gsnap (Wu and Nacu 2010), allowing for the discovery of novel splice sites (*i.e.*, deletion break points, –novel-splicing = 1). We retained all detected deletion breakpoints, regardless of their inferred probability of as a donor or acceptor site for mRNA splicing. The same sequencing reads were then aligned to the full-length consensus by TopHat (Trapnell *et al.* 2012), guided by the detected breakpoints from each individual genome and the previously reported breakpoints for *KP* element (Black *et al.* 1987).

In a second approach, which we employed for *P*-elements only, we aligned reads to a genome index that included the *P*-element consensus and the deletion breakpoints for the *KP* element using STAR (Dobin *et al.* 2013). For the first pass, noncanonical and canonical splice junctions were weighted with equal probabilities (–outSJfilterCountUniqueMin 1 1 1 1–outSJfilterOverhangMin 12 12 12 12–outSJfilterCountTotalMin 1 1 1 1–scoreGapNoncan 0–scoreGapGCAG 0–scoreGapATAC 0). The novel deletion breakpoints that were identified, together with the *KP* breakpoints, were used to generate a new genome index for the second pass of STAR alignment.

In the third approach, which we employed for both *P*-elements and *hobo* elements, reads were aligned to the full-length consensus using HISAT2 (Kim *et al.* 2015). There were no penalties for novel breakpoints introduced in either set of alignments, regardless of whether they corresponded to canonical or noncanonical splice sites (–pen-noncansplice 0–pen-cansplice 0–pen-canintronlen G,0,0–pen-noncanintronlen G,0,0). For *P*-elements, two sets of alignments were generated, one where the *KP* deletion breakpoints were provided during the alignment process and one where they were not. The second set of alignments without the *KP* deletion breakpoints were generated because we observed that the breakpoints prevented the detection of the *KP* variant by the assembler StringTie (Pertea *et al.* 2015).

We applied the Cufflinks (Trapnell *et al.* 2012) and StringTie (Pertea *et al.* 2015) transcript assemblers to each of the three sets of alignments described above. To be included in the assembly, we required that a structural variant must be at least 1% as abundant as the most common structural variant in the library (-F 0.01, Cufflinks; -f 0.01 StringTie) (Trapnell *et al.* 2012). Estimates of relative and absolute abundance of individual structural variants were obtained from their reported coverage in each of the six transcript assemblies. The overall copy number of *P*-elements and *hobo* elements (regardless of structural variation) was estimated by dividing the summed coverage across all structural variants in the sequencing library by the average coverage for that same library across the reference genome (Table S1 and Table S2). The number of full-length elements was estimated by dividing the coverage of the full-length element by the average coverage across the reference genome (Table S1 and Table S2). For *P*-elements only, the relative abundance of full-length elements, *KP* elements, and other deletion derivatives, was estimated as the proportion of coverage across all structural variants that corresponded to each variant type (Table S3). The ratio of full-length to *KP* elements (*FP*/*KP* ratio) was estimated as the coverage ratio for these two structural variants (Table S3). A constant of 0.01 was added to both the full-length and *KP* coverage values when calculating the *FP*/*KP* ratio to avoid producing zero and nonexistent values.

### Fly stocks

DGRP lines were obtained from the Bloomington *Drosophila* Stock Center. Canton-S is a reference susceptible stain (“M” strain) (Kidwell and Novy 1979; Schaefer *et al.* 1979) that was kindly provided by Richard Meisel.

### Paternal induction of hybrid dysgenesis

Canton-S virgin females were crossed to males from different DGRP lines, and their F1 females were collected and aged for 3 d. To detect hybrid dysgenesis, we examined 3-d-old F1 females for the presence of rudimentary atrophied ovaries (Schaefer *et al.* 1979). In reference dysgenic crosses, the incidence of ovarian atrophy is temperature dependent, with 100% of F1 females exhibiting atrophied ovaries at 29°, but <5% of F1 females exhibiting atrophied ovaries at 18° (Kidwell *et al.* 1977; Kidwell and Novy 1979; Engels and Preston 1979). Therefore, we maintained our crosses and the resulting F1 offspring at 25°, where the intermediate incidence of hybrid dysgenesis would maximize the potential to detect differences between strains. We performed our experiments in nine experimental blocks, where each DGRP line was randomly assigned to two blocks.

Ovarian atrophy was assayed with a squash prep (Simmons *et al.* 2007). Briefly, each female was squashed under a coverslip with a few drops of food color solution, which facilitates the visualization of developing egg chambers. Females lacking egg chambers were scored as exhibiting atrophied ovaries, while females harboring egg chambers were scored as exhibiting nonatrophied ovaries. Of the 52 DGRP lines that were considered, 33 produced at least 20 F1 females in two separate experimental blocks. Only these 33 lines were included in the regression analysis (Table S4).

### Regression analysis

The presence or absence of atrophied ovaries in 1969 individual F1 female offspring from 33 different DGRP lines was modeled with mixed effects logistic regression. Logistic regression is the preferred approach for examining the relationship between a numeric variable (*P*-element abundance or *hobo* abundance) and a binomial outcome (presence or absence of developing egg chambers). Models were fitted using the glmer function of the lme4 package in R (Bates *et al.* 2015). To control for potential environmental effects on oogenesis and ovarian atrophy, such as variation in food quality (Drummond-Barbosa and Spradling 2001) and temperature fluctuations (Kidwell *et al.* 1977; Kidwell and Novy 1979; Engels and Preston 1979), all models included experimental block as a random effect. Some models also included paternal DGRP line as a random effect.

### PCR

Genomic DNA was extracted from selected DGRP genomes using squish prep. 5–30 adult flies were homogenized in 100 μl of solution containing 100 mM Tris-HCl (pH 7.5), 100 mM EDTA, 100 mM NaCl, and 1% SDS, and incubated for 30 min at 65°. Following the addition of 180 μl of a solution of 1.43 M KAc and 4.29 M LiCl, tubes were incubated for 10 min on ice. The homogenate was centrifuged for 15 min at >12,000 × *g* (4°) and DNA was precipitated from the resulting supernatant with 120 μl of isopropanol. Pellets were rinsed in 70% ethanol and resuspended in ddH_2_O.

To explore the range of *P*-element structural variants in DGRP genomes, we employed a primer that will anneal to any full-length element with a pair of intact terminal inverted repeats (TIRs) (Rasmusson *et al.* 1993). Different deletion derivatives produce amplicons of different sizes, and long, 3 min extension times were used to enrich for longer deletion derivatives (Hill *et al.* 2016). We also used a PCR assay specific to the *KP* elements, to curate the presence and absence of this deletion derivative (Rasmusson *et al.* 1993). Canonical *hobo* elements were similarly amplified using primers and conditions described previously (Deprá *et al.* 2009).

### Data availability

Ovarian atrophy scores used for logistic regression analysis, as well as the corresponding paternal strain and experimental block, can be found in Table S4.

## Results

### DGRP lines exhibit heritable variation in paternal induction of hybrid dysgenesis

**Figure 1:**
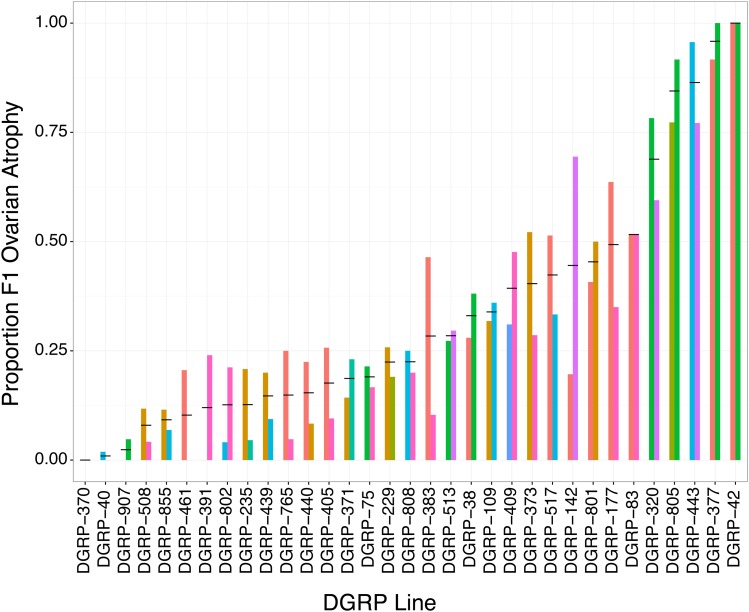
Variation paternal in induction of hybrid of dysgenesis among DGRP lines. Thirty-three DGRP lines are indicated on the *x*-axis. The *y*-axis indicates the proportion of F1 offspring that exhibited ovarian atrophy in crosses between the DGRP males and Canton-S females. Bars are colored according to experimental block. Lines are sorted according to the average proportion of F1 females exhibiting atrophied ovaries across both experimental blocks, which is indicated by the back horizontal bar. DGRP, *Drosophila* Genetic Reference Panel.

To uncover variation in paternal induction of hybrid dysgenesis among DGRP lines, we crossed DGRP males to females from Canton-S, a reference strain that does not repress *P*-element activity (Kidwell *et al.* 1977). Among the 33 different DGRP lines that we successfully assayed in two biological replicates, we observed dramatic variation in the incidence of hybrid dysgenesis, with the proportion of F1 females exhibiting atrophied ovaries ranging from 0 to 1 (Figure 1). To determine whether hybrid dysgenesis is associated with the paternal (DGRP) genotype, we compared a null logistic regression model that included only experimental block, to an alternative model that also included the paternal DGRP strain. The alternative model explained significantly more variation in the incidence of F1 ovarian atrophy, as indicated by a dramatic drop in the Akaike Information Criterion (AIC) Score (ΔAIC = −420.18) and a highly significant drop in deviance test (χ^2^ = 484.18, d.f. = 1, *P* < 3.42e−82). Therefore, the 33 DGRP lines we examined exhibit significant heritable variation in paternal induction of hybrid dysgenesis.

### P-element copy numbers vary among DGRP genomes

**Figure 2:**
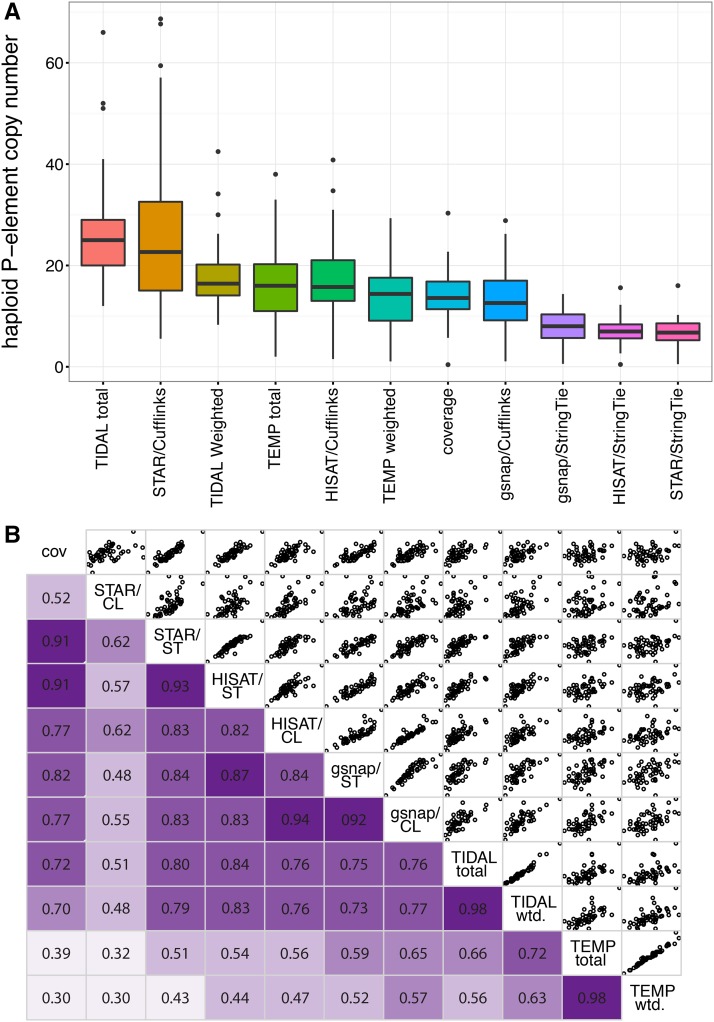
*P*-element abundance varies among DGRP genomes. (A) Distribution of haploid copy numbers among 52 genomes is shown for 11 different estimators of *P*-element abundance. (B) Correlation matrix of haploid copy numbers from each of the 11 estimators. In the lower panels, Pearson’s *R* correlation values are given, with darker shades of purple indicating stronger correlations. The upper panel provides scatter plots comparing pairs of estimators. CL, Cufflinks; cov, normalized coverage; DGRP, *Drosophila* Genetic Reference Panel; ST, StringTie; wtd., weighted.

We next looked for variation in *P*-element abundance among DGRP genomes, which could explain differences in paternal induction of hybrid dysgenesis. For 52 DGRP lines, we considered eleven different estimates of overall *P*-element abundance: the normalized *P*-element sequencing coverage, the total number of annotated insertions and the estimated average copy number based on TIDAL and TEMP annotations, and the estimated copy numbers based on six different assemblies of *P*-*element* structural variants in each DGRP genome (see *Materials and Methods*, Figure 2A, Table S1, and Table S2). All eleven estimators suggest varied *P*-element abundance among DGRP genomes (Figure 2A), and abundances estimated by different methods are generally highly correlated (Figure 2B).

There are systematic differences among the estimators of *P*-element abundance we considered (Figure 2A), with TIDAL total indicating the highest haploid copy number (average 26.2 copies/genome, range: 12–66) and STAR/StringTie assembly indicating the lowest (average 6.83 copies/genome, range: 0.53–16.01) (Figure 2, A and B and Table S1). Depending on the estimator, our observations are consistent with—or modestly lower than—previous estimates of *P*-element abundance in wild-derived genomes based on *in situ* hybridization [∼30 copies (Ronsseray *et al.* 1989)], and pooled deep sequencing [∼60 copies (Kofler *et al.* 2015)].

### P-element structural variation among DGRP genomes

**Figure 3:**
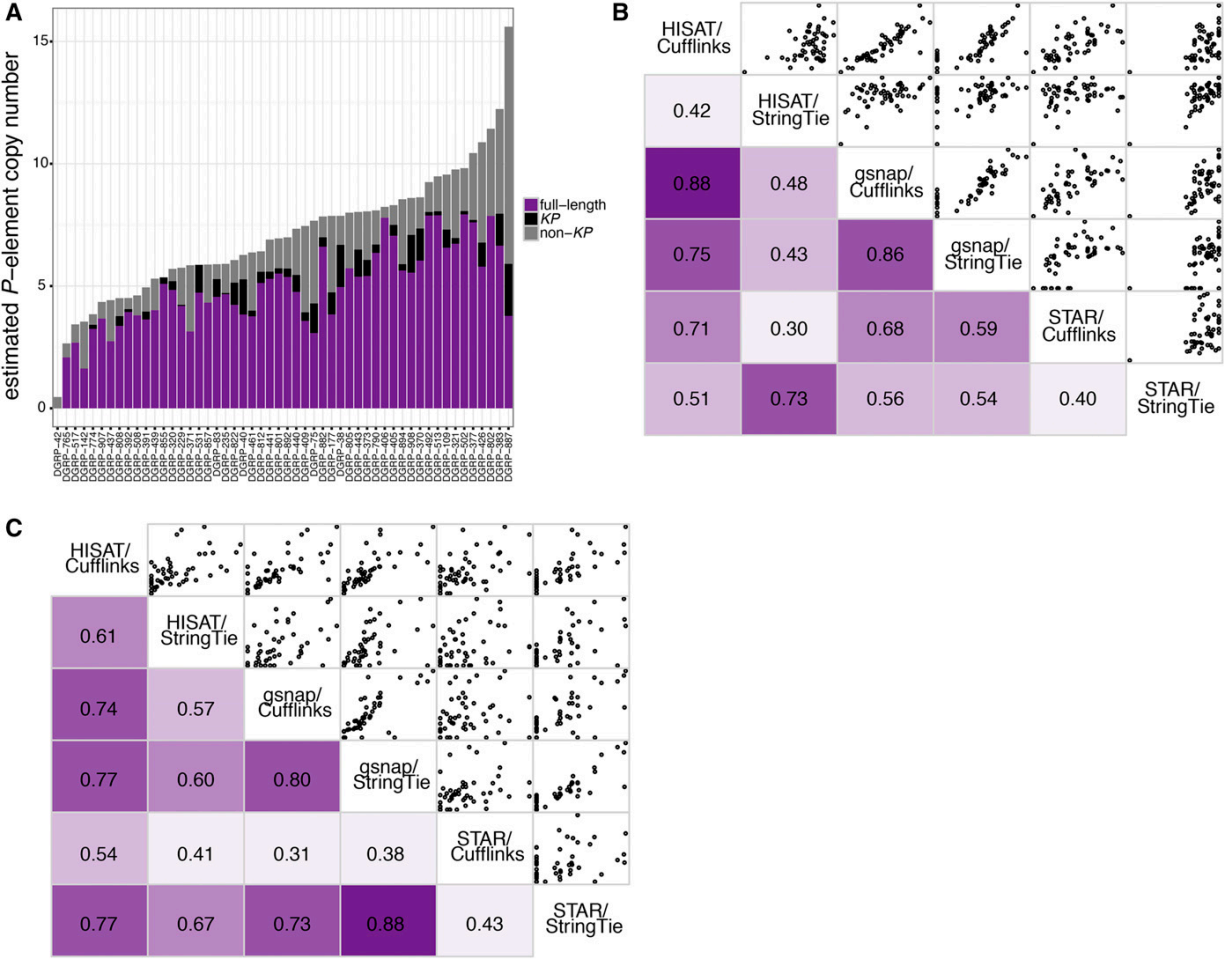
Structural variation among *P*-elements in DGRP genomes. (A) Estimated haploid *P*-element copy numbers for 52 DGRP genomes based on HISAT/StringTie assembly of individual structural variants. The proportion of genomic *P*-elements corresponding to full-length elements, *KP* elements, and non-*KP* deletion derivatives are indicated in purple, black, and gray, respectively. (B and C) Correlation matrix of estimated haploid numbers of full-length (B) and *KP* (C) elements from six different assemblies. In the lower panels, Pearson’s *R* correlation values are given, with darker shades of purple indicating stronger correlations. The upper panel provides scatter plots comparing pairs of estimators. DGRP, *Drosophila* Genetic Reference Panel.

To identify structural and functional variation among *P*-elements in DGRP genomes, which may explain differences in paternal induction of hybrid dysgenesis (Figure 1), we estimated the abundance of full-length *P*-elements, *KP* deletion derivatives, and non-*KP* deletion derivatives using software designed to detect and quantify isoforms in RNA-seq data (Figure 3A, see *Materials and Methods*). To evaluate the accuracy of each of six different *P*-element structural variant assemblies we generated, we compared the presence and absence of *KP* elements in the assembly to the presence or absence of *KP* elements in samples of genomic DNA (Table S5). *KP* elements are common in natural populations, and their presence or absence is easily detected by PCR with breakpoint-specific primers (Rasmusson *et al.* 1993). Out of 18 DGRP genomes we examined via PCR, all six assemblies correctly identified the presence or absence of *KP* elements significantly more often than expected by chance, with the HISAT/StringTie assembly performing the best (correct presence/absence in 17 out of 18 genomes, Table S5). Furthermore, the abundance of full-length and *KP* elements among DGRP genomes is correlated between all pairs of assemblies (Figure 3, B and C). These results suggest that isoform assembly software is able to detect and quantify TE deletion derivatives from deep sequencing data.

According to the results from HISAT/StringTie, the proportion of *P*-element copies that are full-length varies considerably among the 52 DGRP genomes, ranging from 0 (DGRP-42) to 0.95 (DGRP 855, Figure 3A and Table S2). *KP* elements were detected in 39 out of the 52 DGRP genomes, consistent with previous reports that they are common among North American *D. melanogaster* (Itoh *et al.* 2007). The estimated proportion of genomic *P*-elements that corresponds to the *KP* variant was also highly variable between strains, ranging from 0 to 0.23.

### The relationship between hybrid dysgenesis and paternal P-element dosage is weak

We next combined our phenotype and genotype data to determine whether increasing paternal *P*-element dosages is associated with hybrid dysgenesis. We compared full mixed-effect logistic regression models containing an estimator of *P*-element abundance to reduced models that lack the abundance estimators (Table 2). Surprisingly, for 8 out of 11 abundance estimators, we observed that the full model did not provide a better fit than the reduced model, indicating that differences in paternal *P*-element dosage are not associated with different odds of hybrid dysgenesis among female offspring (Table 2 and Table S6). More unexpectedly, the remaining three estimators of *P*-element abundance were negatively associated with ovarian atrophy, indicating that fathers with more *P*-elements were less—not more—likely to produce dysgenic offspring.

**Table 2:** Logistic regression of *P*-element copy number and structural variation with hybrid dysgenesis

*P*-Element Estimate	*P*-Value (Full Data Set)	Association (Full Data Set)	*P*-Value (No DGRP-42)	Association (No DGRP-42)
Normalized coverage	3.55E−01	−	2.28E−05	+
TIDAL-total	2.03E−01	+	4.81E−02	+
TIDAL-weighted	2.81E−01	+	6.59E−05	+
TEMP-total	5.51E−01	+	6.36E−01	+
TEMP-weighted	7.99E−01	−	2.88E−01	−
HISAT/Cufflinks CN	9.36E−01	−	1.22E−05	+
HISAT/StringTie CN	3.49E−01	+	7.13E−04	+
gsnap/Cufflinks CN	5.81E−01	−	4.48E−05	+
gsnap/StringTie CN	3.54E−02	−	1.86E−02	+
STAR/Cufflinks CN	4.72E−06	−	2.67E−03	−
STAR/StringTie CN	4.53E−04	−	7.29E−01	+
HISAT/Cufflinks FL-CN	2.78E−05	−	6.94E−01	−
HISAT/StringTie FL-CN	9.20E−09	−	8.18E−05	−
gsnap/Cufflinks FL-CN	7.33E−02	−	1.75E−01	+
gsnap/StringTie FL-CN	8.60E−01	−	6.44E−01	+
STAR/Cufflinks FL-CN	4.49E−04	−	6.03E−01	−
STAR/StringTie CN	2.32E−04	−	8.44E−01	+
HISAT/Cufflinks FP/KP	2.88E−02	−	2.35E−01	−
HISAT/StringTie FP/KP	1.88E−02	−	6.16E−01	−
gsnap/Cufflinks FP/KP	6.79E−02	−	4.23E−01	−
gsnap/StringTie FP/KP	9.28E−01	+	6.51E−01	−
STAR/Cufflinks FP/KP	4.42E−01	+	3.21E−01	+
STAR/StringTie FP/KP	6.19E−01	−	8.51E−01	−

The fit of alternative models, which includes the indicated estimate of *P*-element abundance or structural variation as a fixed effect, were compared to nested null models. The *P*-values reported result from a likelihood ratio test comparing the two model fits, with a value of <0.05 indicating that the model including an estimate of *P*-element abundance or structural variation provided a significantly better fit to the data than the null model. For each model, the direction of the association between paternal *P*-elements and hybrid dysgenesis is also indicated (±). All model pairs were fitted to both the full data set and the data set excluding the outlier strain DGRP-42. Complete information about model components, fit, and comparison can be found in Table S6. CN, copy number; FL, full-length; FP, full-length *P*-element.

A potential explanation for the weak or negative relationship between paternal copy number and hybrid dysgenesis is that functional differences among *P*-elements in DGRP genomes might confound strict dosage dependence. In particular, full-length elements that encode transposase might contribute disproportionately to dysgenesis, while *KP* elements might offset the effects of full-length copies by encoding repressor proteins. Therefore, we considered whether the abundance of full-length *P*-elements (Table S1), or the ratio of full-length to *KP* elements (*FP*/*KP* ratio, Table S3), might better explain differences in hybrid dysgenesis. However, similar to our observations with overall abundance, we again observed only nonsignificant or negative relationships.

**Figure 4:**
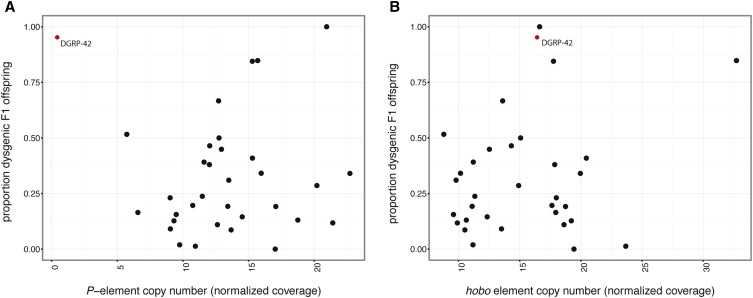
Genotypic and phenotypic associations with F1 hybrid dysgenesis. The proportion of dysgenic offspring of 33 DGRP lines is represented as a function of *P*-element (A) and *hobo* element (B) copy number. TE copy numbers are estimated from coverage over the consensus sequence, normalized to the average genome-wide coverage from the same sequencing library. The influential outlier DGRP-42 is indicated in red. DGRP, *Drosophila* Genetic Reference Panel; TE, transposable element.

The negative association between paternal *P*-elements and F1 hybrid dysgenesis is explained by an influential outlier: DGRP-42 (Figure 4A). Although the DGRP-42 genome harbors relatively few *P*-elements, all of which are internally deleted and therefore do not encode transposase (Figure 3A), it induced ovarian atrophy in 100% of F1 female offspring (Figure 1). When DGRP-42 is excluded from the analysis, 7 out of 11 estimators of *P*-element abundance exhibit a positive and significant association with the odds of hybrid dysgenesis (Table 2 and Table S6). The only *P*-element abundance estimate that remains significantly negatively associated with dysgenesis in the absence of DGRP-42 is STAR/Cufflinks, which we determined was the least accurate assembly (Table S5).

We verified that the capacity of DGRP-42 to induced ovarian atrophy is not the result of a Mendelian genetic factor by crossing DGRP-42 females to Canton-S males. 100% (20/20) of the F1 females we examined, which were genetically identical to those of the reciprocal experimental cross, exhibited normal ovaries. We further verified that that the unusual behavior of DGRP-42 is not the result of stock contamination in two ways. First, we confirmed the presence of a combination of rare SNPs on the second chromosome that are uniquely diagnostic of DGRP-42. Second, using primers that anneal to the *P*-element TIRs (Rasmusson *et al.* 1993), we examined the size classes of *P*-elements that amplify from DGRP-42 genomic DNA (Figure S1). A single dominant fragment of ∼700 bp was observed, which is consistent with our previous inference that *P*-elements in DGRP-42 are overwhelmingly or exclusively deletion derivatives that are <1 kb in length (Table S3).

### Hobo-elements are also associated with paternal induction of hybrid dysgenesis

In light of our observations with DGRP-42, we considered whether another TE family might contribute to the hybrid dysgenesis observed among the offspring of DGRP males and Canton-S females. In particular, ovarian atrophy can also occur in the *hobo* element dysgenesis syndrome (Yannopoulos *et al.* 1986, 1987; Blackman *et al.* 1987). In addition to lacking *P*-elements, many sublines of Canton-S lack canonical *hobo* elements (*hobo*-empty) and produce dysgenic offspring in crosses with males from certain strains bearing active *hobo* copies (H strains, Blackman *et al.* 1987; Pascual and Periquet 1991; Bonnivard *et al.* 1997; Rahman *et al.* 2015). We confirmed via PCR that canonical *hobo* elements are absent from our Canton-S subline, indicating that they are *hobo*-empty (Figure S2). In contrast, PCR suggests canonical full-length *hobo* elements are present in DGRP-42 (Figure S2), consistent with the dysgenesis syndrome we observe in their F1 offspring.

**Figure 5:**
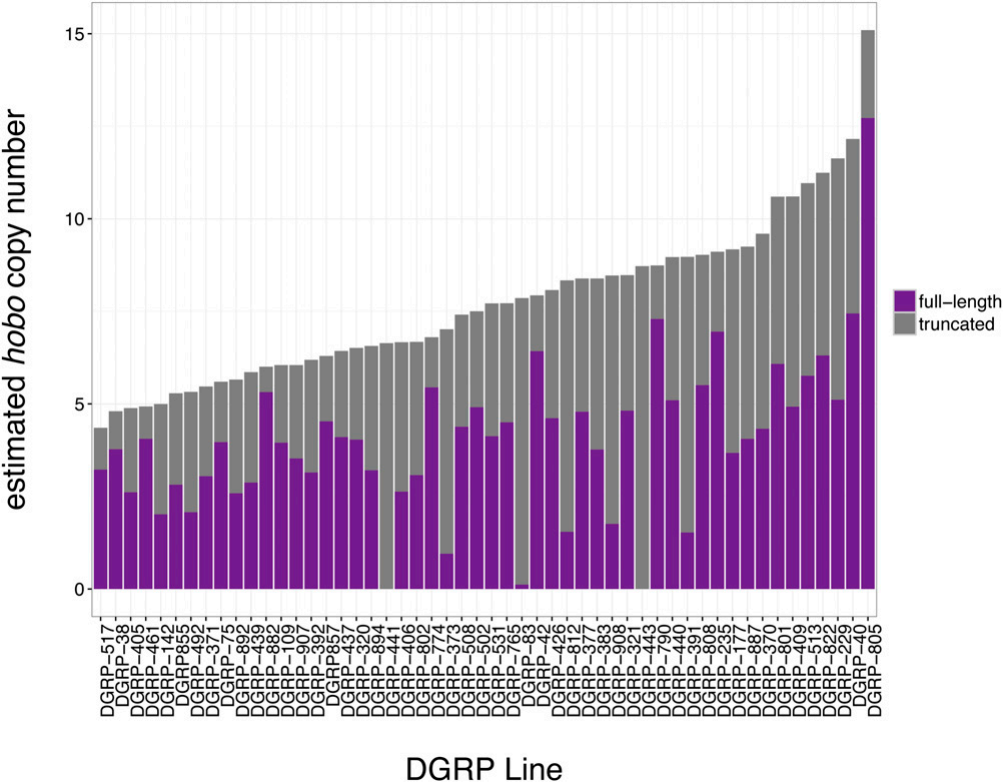
Structural and copy number variation in *hobo*-elements in DGRP genomes. Estimated haploid *hobo* element copy numbers for 52 DGRP genomes based on HISAT/StringTie assembly of individual structural variants. The proportion of genomic *hobo* elements corresponding to full-length elements, and deletion derivatives are indicated in purple and gray, respectively. DGRP, *Drosophila* Genetic Reference Panel.

We next considered whether paternal *hobo* element abundance might be associated with paternal induction of hybrid dysgenesis among the 33 DGRP lines we assayed. Using the same approaches that we developed for *P*-elements, we estimated *hobo* element abundance in 52 DGRP genomes (Figure 5). Notably, DGRP-42 is estimated to have the third largest number of full-length *hobo* elements of the 33 DGRP genomes that we assayed for induction of hybrid dysgenesis (Figure 5 and Table S2). We examined the relationship between *hobo* abundance and hybrid dysgenesis by comparing nested logistic regression models. The normalized *hobo* element coverage significantly improved the fit of a logistic regression model when compared to the null model (−2ΔLnL = 15.07, d.f. = 1, *P* = 1.04E−11, Table S7). Indeed, based on the AIC score, *hobo* coverage is a more powerful predictor of the odds of hybrid dysgenesis than *P*-element abundance (Table S6 and Table S7). The estimated number of full-length *hobo*-elements, based on a HISAT/StringTie assembly of structural variants, also significantly improved the model fit (−2ΔLnL = 7.18, d.f. = 1, *P* = 7.37E−3, Table S7). Consistent with a dosage-dependent relationship, both normalized *hobo* coverage and StringTie-estimated full-length copy numbers are positively associated with the odds of F1 hybrid dysgenesis (Figure 4B and Table S7).

Given our observations that both *hobo* and *P*-element abundance are independently associated with hybrid dysgenesis, we examined models that include abundance estimators of both transposons. We compared a reduced model that included only experimental block and *hobo* coverage to a series of models containing estimators of *P*-element abundance. We observed that the addition of any of four (out of 11) estimators of overall *P*-element abundance, three (out of six) estimators of full-length *P*-element abundance, and two (out of six) estimators of *FP/KP* ratio significantly improve model fit (Table 3 and Table S8). However, with two exceptions (TIDAL total and STAR/Cufflinks *FP/*KP), *P*-elements are negatively—not positively—associated with the odds of hybrid dysgenesis in the full model. As with our models that considered *P*-elements only (Table 2 and Table S6), these negative associations are almost entirely dependent on DGRP-42. When DGRP-42 is excluded from the analysis, six (out of 11) estimators of overall *P*-element abundance are significantly and positively associated with hybrid dysgenesis (Table 3). Only a single negative association between the abundance of full-length elements and hybrid dysgenesis remains significant, with an increased *P*-value. Therefore, DGRP-42 remains an influential outlier in the relationship between genomic *P*-elements and hybrid dysgenesis even after differences in *hobo* abundance are considered.

**Table 3:** Logistic regression of *P*-element copy number and structural variation with hybrid dysgenesis after accounting for differences in *hobo*-abundance

*P*-*element* Estimate	*hobo* Estimate	*P*-Value (Full Dataset)	Association (Full Dataset)	*P*-Value (No DGRP-42)	Association (No DGRP-42)
Normalized coverage	Normalized coverage	2.73E−01	−	8.59E−05	+
TIDAL-total	Normalized coverage	5.03E−03	+	2.17E−07	+
TIDAL-weighted	Normalized coverage	2.12E−01	+	4.75E−05	+
TEMP-total	Normalized coverage	2.83E−01	+	4.97E−01	+
TEMP-weighted	Normalized coverage	9.28E−01	−	3.66E−01	−
HISAT/Cufflinks CN	Normalized coverage	5.63E−01	−	2.15E−04	+
HISAT/StringTie CN	Normalized coverage	3.74E−01	−	9.43E−04	+
gsnap/Cufflinks CN	Normalized coverage	3.32E−01	−	3.44E−04	+
gsnap/StringTie CN	Normalized coverage	1.34E−02	−	5.96E−02	+
STAR/Cufflinks CN	Normalized coverage	7.37E−05	−	1.19E−02	−
STAR/StringTie CN	Normalized coverage	3.61E−04	−	8.72E−01	+
HISAT/Cufflinks FL-CN	Normalized coverage	3.99E−05	−	6.77E−01	−
HISAT/StringTie FL-CN	Normalized coverage	5.10E−07	−	8.41E−04	−
gsnap/Cufflinks FL-CN	Normalized coverage	1.03E−01	−	1.47E−01	+
gsnap/StringTie FL-CN	Normalized coverage	3.21E−01	−	1.68E−01	+
STAR/Cufflinks FL-CN	Normalized coverage	1.32E−03	−	7.46E−01	−
STAR/StringTie FL-CN	Normalized coverage	3.65E−04	−	8.54E−01	+
HISAT/Cufflinks FP/KP	Normalized coverage	2.23E−01	−	7.24E−01	−
HISAT/StringTie FP/KP	Normalized coverage	1.02E−02	−	4.57E−01	−
gsnap/Cufflinks FP/KP	Normalized coverage	7.46E−02	−	4.47E−01	−
gsnap/StringTie FP/KP	Normalized coverage	9.79E−02	−	6.16E−01	−
STAR/Cufflinks FP/KP	Normalized coverage	1.06E−06	+	7.75E−08	+
STAR/StringTie FP/KP	Normalized coverage	7.08E−01	−	2.54E−01	+

The fit of null models that include *hobo* abundance as a fixed effect and experimental block as a random effect was compared to alternative models that also include an estimate of *P*-element abundance or structural variation. The *P*-values reported result from a likelihood ratio test comparing the two model fits, with a value of <0.05 indicating that the model including an estimate of *P*-element abundance or structural variation provided a significantly better fit to the data than the null model. For each model, the direction of the association between paternal *P*-elements and hybrid dysgenesis is also indicated (±). All model pairs were fitted to both the full data set and the data set excluding the outlier strain RAL-42. Complete information about model components, fit, and comparison can be found in Table S8. CN, copy number; FL, full-length; FP, full-length *P*-element.

## Discussion

Our examination of paternal induction of F1 ovarian atrophy by 33 DGRP strains uncovered heritable phenotypic variation in the fitness costs that are imposed by paternally inherited TEs (Figure 1). Previous studies suggest that these costs reflect the activity of paternally-inherited *P*-elements (Kidwell 1983; Itoh *et al.* 2007), which are unregulated in the F1 offspring due to the absence of maternally-transmitted *P*-element derived piRNAs from the Canton-S mothers (Brennecke *et al.* 2008; Khurana *et al.* 2011). Although *P*-elements are unequivocally a causative agent of hybrid dysgenesis (Bingham *et al.* 1982; Daniels *et al.* 1987; Hill *et al.* 2016), we observed that variation in the odds of ovarian atrophy among the F1 offspring of DGRP males and Canton-S females was only weakly related to the paternal dosage of *P*-elements (Figure 4A, Table 2, and Table S6). Our findings are consistent with previous studies that have also failed to detect a strong relationship (Bingham *et al.* 1982; Boussy *et al.* 1988), or any relationship (Rasmusson *et al.* 1990; Itoh and Boussy 2002), between *P*-element dosage and paternal induction of hybrid dysgenesis. Given the precise estimates of *P*-element copy number that we were able to obtain from next-generation sequence data (Figure 2), it is unlikely that the absence of strong dosage dependence reflects technological limitations in estimating *P*-element abundance. We also did not observe that the weak relationship could be explained by the presence of deletion derivatives that may titrate transposase encoded by full-length elements (Simmons and Bucholz 1985) or encode repressor proteins (Black *et al.* 1987; Robertson and Engels 1989; Gloor *et al.* 1993; Simmons *et al.* 2002; Jensen *et al.* 2008). Overall, our data suggest that, although the presence of unregulated *P*-elements may induce F1 hybrid dysgenesis, their dosage is not strongly correlated with severity of the dysgenic phenotype.

Interestingly, we observed that *hobo* element abundance was more strongly associated with paternal induction of hybrid dysgenesis in our full data set than *P*-element abundance (Figure 4B, Table S6 and Table S7). Although the relationship between *hobo* elements and ovarian atrophy was still weak, our results suggest that *hobo* elements may contribute to the dysgenesis syndrome that is observed among F1 offspring of Canton-S females and DGRP males. Indeed, the odds of hybrid dysgenesis among F1 females were best predicted by models that considered the paternal abundance of both *P*-elements and *hobo* elements (Table S8). These results are unexpected, since *hobo* inducer strains have been quite rare in previous surveys of natural populations (Pascual and Periquet 1991; Bonnivard *et al.* 1997). Indeed, most studies that have examined hybrid dysgenesis in the offspring of Canton-S females mated to recently collected male genotypes, many of which were published before *hobo* elements were characterized (Blackman *et al.* 1987; Yannopoulos *et al.* 1987), assume that paternally inherited *P*-elements are the only causative agents (Kidwell 1983; Anxolabéhère *et al.* 1988; Boussy *et al.* 1988; Itoh *et al.* 2001, 2004; Itoh and Boussy 2002; Ogura *et al.* 2007; Onder and Kasap 2014; Ignatenko *et al.* 2015). Therefore, although studies in which laboratory populations are infected with *P*-elements unequivocally demonstrate that paternal *P*-elements can induce hybrid dysgenesis (Bingham *et al.* 1982; Daniels *et al.* 1987), our observations suggest that paternal induction that is exhibited by many recently collected wild-derived genotypes may reflect the combined action of *P*-elements and *hobo* elements.

Regardless of the relative roles of *P*-elements and *hobo* elements in inducing the dysgenic phenotype, the two TEs together still explain relatively little of the heritable variation that we observed in paternal induction of hybrid dysgenesis. Although it is not possible to quantify how much variation in a binomial outcome (presence or absence of atrophied ovaries) is explained by a particular variable or model, the AIC scores of two models that include different explanatory variables can be compared to determine which factors have the most explanatory power. For the full data set, a model that accounts for heritable variation in the odds of ovarian atrophy by considering paternal DGRP strain as a fixed effect (AIC = 1823.4) provides a dramatically better fit to the data than the best fit model that includes only *P*-element and *hobo* abundance as fixed effects (AIC = 2275.25, Table S8). Therefore, other genetic factors in addition to the *P*-elements and *hobo* elements must have an important role in determining the penetrance of the dysgenic phenotype.

Position effects of particular TE insertions provide one potential source of unexplained heritable variation in paternal induction of hybrid dysgenesis. If individual *P*-elements or *hobo* elements are more likely to induce genome instability through excision or participation in ectopic recombination, then the presence or absence of such elements in a particular genotype would affect the odds of ovarian atrophy among F1 offspring. Unfortunately, such position effects would be difficult to detect, because most *P*-element insertions exist at low frequency in natural populations (Kofler *et al.* 2012), and therefore are only sampled in a few DGRP genotypes (Zhuang *et al.* 2014; Rahman *et al.* 2015). Exceptionally deleterious insertions that increase the fitness costs of hybrid dysgenesis are expected to be particularly rare.

Host-encoded factors that modulate TE activity or TE-associated fitness effects are another potential source of unexplained variation in hybrid dysgenesis. For example, inverted repeat binding protein (IRBP) and its partner Xrp1 bind to the TIRs of *P*-elements, and are thought to facilitate repair after excision (Rio and Rubin 1988; Beall *et al.* 1994; Beall and Rio 1996; Francis *et al.* 2016). Genetic variation in such factors could act as modifiers to a TE-induced dysgenic phenotype. Indeed, proteins that are involved in TE regulation often exhibit signatures of adaptive evolution, such as zinc-finger proteins in mammals (Thomas and Schneider 2011; Jacobs *et al.* 2014) and piRNA effector proteins in mammals and *Drosophila* (Obbard *et al.* 2009; Kolaczkowski *et al.* 2011; Simkin *et al.* 2013), suggesting that they may evolve rapidly to minimize the fitness costs of TE activity. Mapping these host-encoded genetic variants would be an interesting avenue for a future study with a larger sample size.

TE-associated fitness costs, such as hybrid dysgenesis, are considered the major force that opposes the exponential spread of TEs through eukaryotic genomes [reviewed in Charlesworth and Langley (1989) and Nuzhdin (1999)]. In particular, Charlesworth and Charlesworth (1983) determined that TE copies must act in a synergistic and dosage-dependent manner to reduce host fitness in order for negative selection to counterbalance TE proliferation. Our observations support this model in demonstrating weak dosage-dependent fitness effects of two DNA transposons on female fertility. However, we also uncover heritable variation in TE-induced fitness affects that is independent of dosage, implying that other factors, such as host regulation, must also oppose TE proliferation in natural populations.

## Supplementary Material

Supplemental material is available online at www.g3journal.org/lookup/suppl/doi:10.1534/g3.117.040634/-/DC1.
