# Supplementary material for: Paternal Induction of Hybrid Dysgenesis in Drosophila melanogaster Is Weakly Correlated with Both P-Element and hobo Element Dosage
Source: G3 (Bethesda). 2017 Mar 17;7(5):1487–97. doi: 10.1534/g3.117.040634 (PMC5427502; doi:10.1534/g3.117.040634)
Supplement: Supplementary file 1 [file 1487FigureS1.pdf]

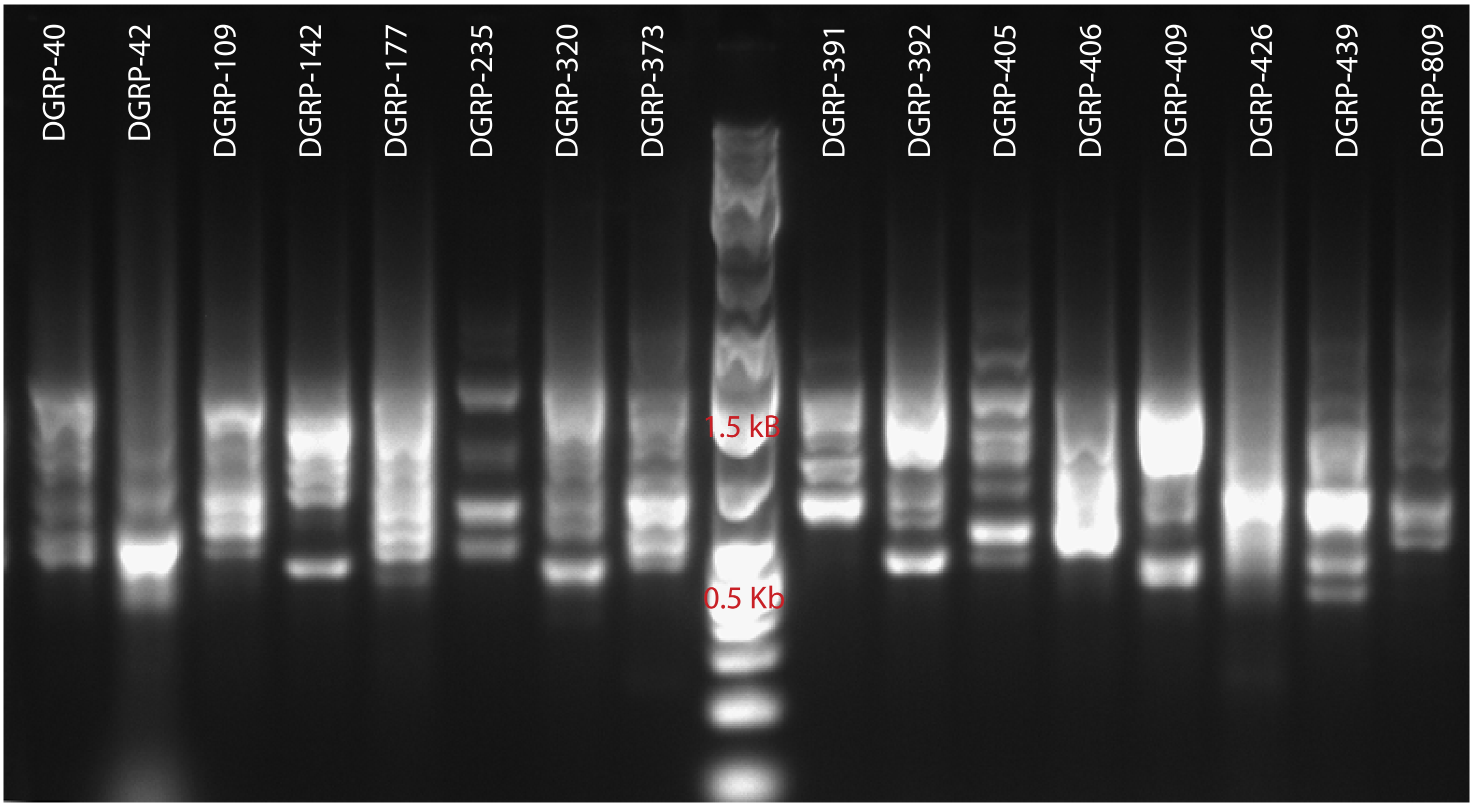

Supplementary Figure 1. Amplification of Genomic *P*-elements from 16 DGRP genomes. PIR primers were used to amplify *P*-elements containing in tact terminal inverted repeats (Rasmusson *et al.* 1993). Most genomes exhibit a range of structural variants. DGRP-42 exhibits only 3 structural variants, all less than 1 kB in length, the most abundant of which is ~0.7 kB.
