# Supplementary material for: Paternal Induction of Hybrid Dysgenesis in Drosophila melanogaster Is Weakly Correlated with Both P-Element and hobo Element Dosage
Source: G3 (Bethesda). 2017 Mar 17;7(5):1487–97. doi: 10.1534/g3.117.040634 (PMC5427502; doi:10.1534/g3.117.040634)
Supplement: Supplementary file 2 [file 1487FigureS2.pdf]

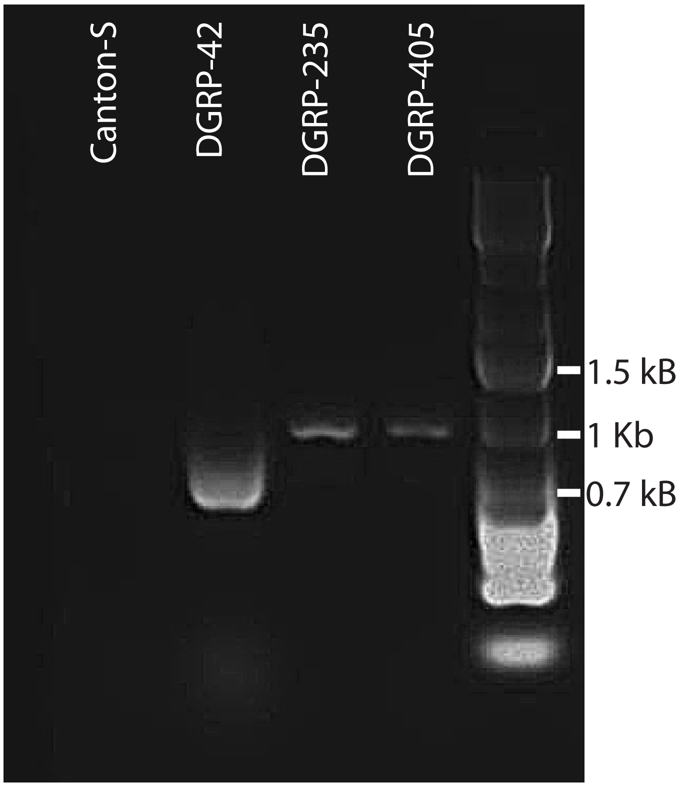

Supplementary Figure 2. Amplification of Genomic *hobo* elements. Hobo-CN primers were used to amplify canonical *hobo* elements (Deprá *et al*. 2009). Fragments amplified from DGRP-42 are the expected size for a full-length element (623 nt). ~ 1kB fragments from DGRP-235 and DGRP-405 correspond to longer structural variants. Canton-S genomic DNA exhibits no canonical *hobo* elements.
